# A comparative effectiveness study of the Breaking the Cycle and Maxxine Wright intervention programs for substance-involved mothers and their children: study protocol

**DOI:** 10.1186/s40359-023-01484-w

**Published:** 2024-01-05

**Authors:** Nicole Racine, Sophie Barriault, Mary Motz, Margaret Leslie, Nancy Poole, Shainur Premji, Naomi C. Z. Andrews, Denise Penaloza, Debra Pepler

**Affiliations:** 1 School of Psychology, University of Ottawa, 136 Jean-Jacques Lussier, Ottawa, ON K1N 6N5 Canada; 2 Children’s Hospital of Eastern Ontario Research Institute, 401 Smyth Road, Ottawa, ON K1H 8L Canada; 3 Mothercraft Breaking the Cycle, 393 King Street East, Toronto, ON M5A 1L3 Canada; 4 Centre of Excellence for Women’s Health, Vancouver, BC V6H 3N1 Canada; 5 Centre for Health Economics, University of York, York, UK; 6 Department of Child and Youth Studies, Brock University, 1812 Sir Isaac Brock Way, St. Catharines, ON L2S 3A1 Canada; 7 Maxxine Wright Community Health Centre, 13733 92 avenue, Surrey, BC V3V 1H9 Canada; 8 Department of Psychology, York University, 4700 Keele Street, Toronto, ON M3J 1P3 Canada

**Keywords:** Mother-child relationship, Early intervention, Substance use, Addiction, Adverse childhood experiences, Quasi-experimental design

## Abstract

**Background:**

Children of substance-involved mothers are at especially high risk for exposure to adverse childhood experiences (ACEs) and poor mental health and development. Early interventions that support mothers, children, and the mother-child relationship have the greatest potential to reduce exposure to early adversity and the mental health problems associated with these exposures. Currently, there is a lack of evidence from the real-world setting demonstrating effectiveness and return on investment for intervention programs that focus on the mother-child relationship in children of substance-involved mothers.

**Methods:**

One hundred substance-involved pregnant and/or parenting women with children between the ages of 0–6 years old will be recruited through the Breaking the Cycle and Maxxine Wright intervention programs, in Toronto, Ontario, Canada and Surrey, British Columbia, Canada, respectively. Children’s socioemotional development and exposure to risk and protective factors, mothers’ mental health and history of ACEs, and mother-child relationship quality will be assessed in both intervention programs. Assessments will occur at three time points: pre-intervention, 12-, and 24-months after engagement in the intervention program.

**Discussion:**

There is a pressing need to identify interventions that promote the mental health of infants and young children exposed to early adversity. Bringing together an inter-disciplinary research team and community partners, this study aligns with national strategies to establish strong evidence for infant mental health interventions that reduce child exposure to ACEs and support the mother-child relationship. This study was registered with clinicaltrials.gov (NCT05768815) on March 14, 2023.

**Supplementary Information:**

The online version contains supplementary material available at 10.1186/s40359-023-01484-w.

## Background

 Exposure to Adverse Childhood Experiences (ACEs), including abuse, neglect, and family dysfunction, is one of the top threats to children’s mental health and development [1, 2]. Children exposed to ACEs are far more likely than children not exposed to experience socioemotional difficulties and post-traumatic stress [3], both of which are precursors to mental illness in adulthood [1, 4–7]. Most children (57.8%) experience at least one ACE in their childhood [8]; however, children exposed to cumulative adversity are at highest risk of poor long-term health and mental health outcomes [1]. Children of substance-involved mothers are at especially high risk for exposure to cumulative ACEs [9] and their developmental consequences, given that maternal substance use often co-occurs with other risk factors, including household violence, intergenerational trauma, poverty, lack of social support, and poor maternal mental health [10]. Exposure to ACEs can thus be intergenerational in nature, whereby mothers who experienced a lack of safety and support in their own childhood struggle to provide nurturance to their children [11–13]. As such, early intervention programs are needed to mitigate poor outcomes for young children of substance-involved mothers.

The first five years of a child’s life establish the building blocks for physical and mental health across the lifespan, with the steepest changes in brain development occurring during this critical period. Exposure to ACEs, particularly in the first five years of life, has a cascading negative impact on brain development [14], increasing the risk for mental health challenges across the lifespan. Thus, intervening to disrupt the negative cascade associated with exposure to ACEs in young children is critical for mitigating poor outcomes and fostering optimal development [15, 16]. It can also attenuate the lag in child development typically observed in children of substance-involved mothers [16]. Additionally, there is growing evidence that interventions during the early years not only benefit families, but also yield long-term economic returns for society [15, 17]. At the population level, it is estimated that a 10% reduction in ACE prevalence could lead to an annual cost savings of up to $105 billion USD and an annual reduction of up to 3 million ACE-attributable disability-adjusted life years [18]. Despite the broad impact of these findings, the mental health of infants, young children, and their families living in contexts of high psychosocial risk has been historically underprioritized [19] suggesting the need for prevention and intervention approaches.

Interventions that simultaneously support mothers, children, and the mother-child relationship have the greatest potential to reduce mental health problems associated with exposure to early adversity [18, 19]. Specifically, interventions that support mothers’ stability by addressing trauma and substance use problems and provide programming related to parenting, healthy relationships, and family violence are the most promising for reducing childhood adversity and promoting optimal child development [20–22]. Currently, there is a lack of knowledge and evidence from real-world settings demonstrating effectiveness and return on investment for intervention programs that focus on the mother-child relationship in children of substance-involved mothers to reduce adversity and improve infant mental health outcomes. The current study aims to address this knowledge gap by comparing the effectiveness of two intervention programs for substance-involved mothers and their young children being delivered in Canada.

### Objective

This paper describes the protocol for a comparative effectiveness study of the Breaking the Cycle (BTC) and Maxxine Wright Community Health Centre (MWCHC) intervention programs offered to substance-involved mothers and their young children (0–6 years). This study will explore the effectiveness of the mother-child focused BTC intervention as compared to the mother-focused program of the MWCHC program. More specifically, the study aims to establish the comparative effectiveness and mechanisms of change of the infant mental health components at BTC, as well as estimate the long-term social return on investment (SROI) of BTC. The key research questions it aims to answer are:

- 1a: Do children at BTC demonstrate enhanced infant mental health compared to children at MWCHC up to 2 years post-intervention?
- 1b: Are decreases in exposure to child ACEs, maternal stress, and mental health symptoms and increases in environment scores, parenting attitudes, and mother-child relationship scores greater among mothers at BTC than at MWCHC between pre-treatment and 24-months after engagement?
- 1c: Are lower exposure to psychosocial risk, lower maternal mental health difficulties, and lower maternal stress at baseline associated with enhanced infant mental health scores over time?
- 2a: Are the associations between treatment dose and infant mental health scores mediated by parenting attitudes and the quality of the mother-child relationship?
- 2b: Does child exposure to psychosocial risk moderate the association between treatment dose and child outcomes?
- 3: What is the estimated long-term SROI of the combined mother-child focus and infant mental health components of the BTC program?

## Methods

### Design

This intervention study is designed as a non-randomized, non-equivalent, comparison study with two equally allocated parallel groups: the BTC intervention group and the MWCHC intervention group. Given that the two intervention programs serve a similar demographic of women, researchers will compare the BTC and MWCHC groups to establish the comparative effectiveness and mechanisms of change of the additional infant mental health component of BTC. The study has been approved by the University of Ottawa Research Ethics Board (REB # H-02-23-8940) and is registered with ClinicalTrials.gov (NCT05768815). All research is performed in accordance with relevant guidelines and regulations. This protocol paper adheres to the SPIRIT guidelines (see Additional file 1).

### Participants

Participants will comprise substance-involved pregnant and/or parenting women with children between the ages of 0–6 years old. We aim to recruit a total of 100 participants: 50 through the BTC intervention and 50 through the MWCHC intervention. To be eligible for the study, participants must: be receiving services at either BTC or MWCHC; have a child under the age of 6 years; and be able to complete the study questionnaires in English. There are no exclusion criteria for participation in this study.

### Recruitment

Mothers will be invited to participate in the study by BTC and MWCHC clinicians providing services at the community-based intervention programs. The clinicians will introduce the research to potential participants within six weeks following their first intake meeting and before receiving any services (e.g., counselling, group participation). Women will be assured that whether they participate or not in the study will not affect the clinical services they receive at either program. This lag time is essential to build trust and rapport with the mothers [23]. Mothers who are interested in participating in the research will be contacted by a community-based researcher (CBR), embedded within each of the intervention programs, who will further explain the research and ask for consent to participate, to be contacted for follow-ups, and to access their clinical file to obtain demographic and other information about psychosocial risk exposure through a retrospective file review. This is a trauma-informed approach to obtaining information whereby participants are not obligated to report information that they have already shared clinically [23].

### Interventions

#### Breaking the Cycle

BTC is a prevention and early intervention program in Toronto, Canada, serving substance-involved pregnant and/or parenting women with children under six years of age. Established by the Canadian Mothercraft Society in 1995, BTC is funded by the Community Action Program for Children and the Canadian Prenatal Nutrition Program through the Public Health Agency of Canada. BTC uses several theoretical frameworks, including developmental theory (i.e., consideration of the combined contributions of both the prenatal and postnatal environments), attachment theory (i.e., child’s cognitive and emotional sense of self and others is developed within the emotional relationship between infants and their primary caregivers), trauma theory (i.e., people who experience trauma are profoundly impacted by those experiences), relational theory (i.e., people grow through relationships with others), and harm reduction (i.e., reducing negative social and/or physical consequences associated with substance use) [24]. BTC provides three components that act synergistically: (a) individualized services for mothers, (b) individualized services for children, and (c) relational interventions that enhance the mother-child relationship and promote infant mental health. Primary services offered include mental health counselling, addictions services, health and medical services, basic needs support, probation and parole services, parenting services, pregnancy outreach program, childcare, and a developmental clinic [24, 25]. In 2004, BTC was recognized by the United Nations as an exemplary program serving pregnant and parenting women who are substance-involved, and their young children [26]. BTC has documented its knowledge creation through evaluation reports [25, 27], scholarly publications [28–30], and a national implementation of its interpersonal violence intervention [31–35].

#### Maxxine Wright

Founded in 2005 and located in Surrey, British Columbia, the MWCHC supports substance-involved women who are pregnant and/or have young children. Women do not need to have children in their care to receive services. MWCHC is a partnership between the Fraser Health Authority, Atira Women’s Resource Society, and the BC Ministry for Children and Family Development. MWCHC addresses maternal substance use and exposure to violence through a multidisciplinary approach. This includes the following philosophical/theoretical approaches: supportive and woman-centered (i.e., creating a safe and supportive environment for women), harm reduction (i.e., recognizing recovery as a long process and a stepping stone toward wellness), trauma-informed (i.e., recognizing that many women have experienced serious trauma), multidisciplinary and wraparound (i.e., providing wraparound health care and social support services to address women’s and children’s needs), health-centered (i.e., providing health care expertise and positive experiences for women and their infants/children), and relational (i.e., focusing on respectful, non-judgmental relationships and compassion) [36]. Primary services offered include addiction counselling, primary health care, and opiod agonist therapy. Within MWCHC, child-focused services are limited to primary health care (e.g., well baby checks, immunizations, referrals for developmental services, growth tracking, and childcare) and do not address the mother-child relationship.

Both the BTC and MWCHC intervention programs are considered integrated maternal substance-use programs in that they offer supports in additional to maternal substance use treatment [36]. Both programs provide services to mothers and their children. For example, both intervention programs have access to childcare, instrumental parenting support, and developmental education. The main difference between the two intervention programs is that BTC uses an infant mental health framework and provides therapeutic supports (e.g., home visitations) to support child development and the parent-child relationship. Given that MWCHC serves a similar demographic of women as BTC but does not focus on infant mental health, they will serve as a comparison site. Table 1 below presents an overview of the services offered by BTC and MWCHC.

**Table 1:** Comparison of services between Breaking the Cycle (BTC) and Maxxine Wright Community Health Centre (MWCHC)

Service feature		Service approach	BTC	MWCHC
Target population	Pregnant or parenting women with substance use difficulties	Mother-focused	Yes (0-6 years)	Yes (0-6 years)
Structure	Ongoing relationship with the child	Mother-focused	Yes	Yes
	Plan to parent	Infant Mental Health	Yes	No
	Comprehensive, wrap-around approach	Mother-focused	Yes	Yes
	Women-centered, trauma-informed	Mother-focused	Yes	Yes
	Infant Mental Health Frameworks	Infant Mental Health	Yes	No
Basic needs support	Food, clothing, transportation	Mother-focused	Yes	Yes
	Childcare	Mother-focused	Yes	Yes
Broader Services	Individual Family Advocacy	Mother-focused	Yes	Yes
	Case Management/Service Coordination	Mother-focused	Yes	Yes
	Primary Health Care Services (mother/child)	Mother-focused	Yes	Yes
Addiction Services	Individual Addictions Services	Mother-focused	Yes	Yes
	Relapse Prevention Group	Mother-focused	Yes	Yes
	Recovery Support Group	Mother-focused	Yes	Yes
	Trauma and Interpersonal Violence Group	Mother-focused	Yes	Yes
Mental Health	Individual Mental Health Counselling	Mother-focused	Yes	Yes
	Individual Trauma Counselling	Mother-focused	Yes	Yes
	Life Skills/Emotion Coping Group	Mother-focused	Yes	Yes
Prenatal Services	Pregnancy Outreach Program	Mother-focused	Yes	Yes
	Prenatal care Services	Mother-focused	Yes	Yes
Instrumental Parenting Support	Basic Parenting support, child management, developmental education	Mother-focused	Yes	Yes
Child Development Supports	Annual Developmental Assessments	Infant Mental Health	Yes	No
	Developmental Screening (annual)	Infant Mental Health	Yes	Yes
	Individualized Developmental Therapy Plan	Infant Mental Health	Yes	No
	Home-visitation focused on child development	Infant Mental Health	Yes	No
	Therapeutic childcare	Infant Mental Health	Yes	No
Mother-child Relationship Support	Individual parenting support/Interaction Guidance	Infant Mental Health	Yes	No
	New Mom’s Support Group (Infant 0-6 months)	Infant Mental Health	Yes	No
	Parent-Child Mother Goose Program	Infant Mental Health	Yes	No
	Learning through Play Group (infant: 6+months)	Infant Mental Health	Yes	No
	Cooking Healthy Together Group	Infant Mental Health	Yes	No
	Parenting Group Support	Infant Mental Health	Yes	Yes

### Primary outcome measures

Informed by the gold standard for assessing and classifying mental health difficulties in the early years (DC: 0–5 [37]), we will use two domains to assess infant/early childhood mental health: (1) socio-emotional functioning and (2) developmental functioning. Measures that provide a diagnosis of mental and physical health problems are not used in the current study due to the infrequency and reliability of these data.

#### Child’s socio-emotional functioning

Mothers will complete the Ages & Stages Questionnaire: Social-Emotional, Second Edition (ASQ-SE-2) [38] version for children. The ASQ-SE-2 measures a child’s self-regulation, compliance, social-communication, adaptive functioning, independence, and relationships. The version used for the present study consists of a total of 23 items using a three-point scale that includes the option for mothers to indicate whether they feel the behavior is of concern. The ASQ-SE-2 shows good psychometric properties and is widely used in early intervention and mental health programs [39].

#### Child’s developmental functioning

Mothers will complete the Ages & Stages Questionnaire, Third Edition (ASQ-3) version [40]. The ASQ-3 assesses a child’s development across five domains: communication, gross motor, fine motor, problem solving, and personal-social skills. The version used for the present study consists of a total of 36 items requiring parents to indicate whether their baby exhibits the behaviour regularly, sometimes, or not yet. The ASQ-3 has strong psychometrics properties and is an internationally used developmental screening measure [41].

### Secondary outcome measures

#### Child’s exposure to risk and protective factors

To assess children’s exposure to risk (e.g., ACEs, poverty, maternal mental illness) and protective factors (e.g., family social support, childcare, community supports), a cumulative measure designed by Bondi et al. (2020) will be used [42]. These measures identify risk and protective factors, including ACEs, for children of substance-involved mothers specifically and were established using data from previous clients at BTC. The measures can be used with children exposed to varying levels of risk and allitems are coded dichotomously (yes or no).

#### Maternal behaviour

To assess the sensitivity of maternal behaviour from a 10-minute in vivo mother-child interaction, the CBRs at each site will use the Maternal Behavior Q-Set (MBQS), Brief Version [43]. The 25 items are centered on parents’ responses to their child’s behaviors and signals. The brief version of the MBQS has been validated with mother-child dyads and has indicated appropriate psychometric characteristics [43, 44].

#### Maternal stress

To assess maternal stress, mothers will complete the Parenting Stress Index (PSI) Short Form [45]. This scale comprises 36 items that are rated on a 5-point Likert scale of agreement. The items are grouped into three domains: parental distress, parent-child dysfunctional interaction, and difficult child, creating a total stress scale when combined. The empirical validity of the PSI Short Form has been well established.

#### Parenting attitudes

To assess parenting attitudes related to expectations, empathy, and discipline, the Adult Adolescent Parenting Inventory, Version 2.0 (AAPI-2) will be used [46]. The AAPI-2 comprises 40 items and five constructs: inappropriate expectations of children, parental lack of empathy towards children’s needs, strong parental belief in the use of corporal punishment, reversing parent-child family roles, and oppressing children’s power and independence.

#### Self-efficacy and satisfaction as a parent

To assess self-efficacy and satisfaction as a parent, the Being a Parent Scale will be used [47]. This tool has 16 items and requires parents to rate whether they feel each item applies to them, using a 6-point Likert scale ranging from 1 (strongly agree) to 6 (strongly disagree).

#### Quality and quantity of stimulation available to the child

To assess the quality and quantity of stimulation available to the child, the Home Observation for Measurement of the Environment (HOME) Inventory, a 45-item questionnaire, will be used [48]. This tool contains six domains (responsivity, acceptance, organization, learning materials, involvement, and variety) and is used by a trained assessor to indicate whether a behaviour is observed or reported by the parent during the home visit.

#### Maternal depression

To assess maternal depression, the Center for Epidemiological Studies Depression Scale (CES-D) [49] will be used. The CES-D contains 20 items and requires individuals to rate how often they experienced symptoms of depression over the past week.

#### Maternal anxiety

To assess anxiety symptoms, the Beck Anxiety Inventory (BAI) will be used [50]. The BAI comprises 21 items and requires individuals to rate how often they experienced symptoms of anxiety in the past week, using a 4-point scale ranging from “Not at all” to “Severely - I could barely stand it”. A total score is obtained by summing all of the items and can be categorized by low anxiety, moderate anxiety, or potentially concerning levels of anxiety.

#### Maternal adverse childhood experiences

To assess maternal ACEs, mothers will retrospectively report on their ACEs prior to 18 years of age using a 10-item questionnaire with “yes/no” questions pertaining to abuse, neglect, and household dysfunction [51].

### Data collection

Informed by our previous longitudinal, multi-informant, and multi-site research with substance-involved mothers, data for the present study will be collected at three time points: pre-intervention, 12-, and 24-months after engagement in services. This longitudinal design will assess whether infant mental health improves and is sustained through a two-year period. The length of engagement in each of the intervention programs varies (mean length is 18 months with a range of 4 to 71 months) [52]. As such, some mothers will remain in the program for the duration of the study while others will not. The evaluation of primary and secondary outcomes will be similar for BTC and MWCHC, the only differences being that the HOME Inventory will not be applied at MWCHC. Data will be collected by the clinicians providing services onsite and by one CBR embedded at each site. The mothers’ clinical files will be reviewed by the CBRs to gather demographic data, addiction information, trauma and psychosocial risk information, treatments accessed, and treatment duration via retrospective file review. The CBRs will receive training on how to administer the MBQS and will be responsible for the in vivo observations and coding of the mother-child relationships. Participants who are no longer receiving services will be offered the opportunity to return to the intervention program for subsequent data collections, to complete the questionnaires over the phone, or to meet at a neutral location. Mothers will receive compensation for their time in the form of gift certificates to grocery stores at each time point.

### Data management

The CBRs will be responsible for collecting the paper questionnaires and entering the data electronically on the BTC and MWCHC servers. These servers will be used to store the research data as they are secure and regularly used to provide confidential and secure clinical services. All electronic files will be password protected and the paper copies of the questionnaires will be stored in a locked filing cabinet at BTC and MWCHC. Once data collection is complete, the data will be shared with the research team and stored at the University of Ottawa for the analysis period. The data will remain on the University of Ottawa secure servers in a locked laboratory facility on the university’s campus. All computers in the laboratory are kept behind locked doors and have secure login requirements. During the storage period, investigators on the research team will have access to the data. As well, research assistants working within the labs of the investigators will have access to data to assist with analyses and contribute to any research dissemination activities. The data and research documents will be retained for 10 years after the completion of the study.

### Statistical analysis

All data analyses will be conducted in SAS and Mplus. For research question 1a, linear mixed models will be used to model the change in each of the primary outcomes from baseline to 24-months post-engagement for the BTC and MWCHC groups, controlling for demographic variables that potentially differ at baseline (e.g., maternal age, socioeconomic status, maternal ACEs). Slopes of the two groups will be compared using an interaction term. Linear mixed models can accommodate participants with missing observations [20]. For research question 1b, we will analyze secondary outcomes with a similar approach. For research question 1c, we will conduct regression analyses to predict which risk and protective factors identified at baseline predict change in the primary outcome for the BTC group.

For research question 2, we will conduct a series of mediation (indirect effects) and moderator analyses (interaction effects) for the BTC group, with time in treatment as the independent variable and infant mental health outcomes at 24 months as the dependent variable. Mediator (i.e., maternal behaviour, parenting attitudes, and maternal stress) and moderator (i.e., psychosocial risk) variables at 12 months will be used in all analyses.

For research question 3, we will adopt international guidelines for SROI [53]. SROI comprises a structured framework to identify, measure, and value the wider societal impacts of an intervention. [53] To support this work, a four-step approach will be adopted: first, using comparative effectiveness evidence from research question 1, information on key mechanisms of change from research question 2, and in partnership with the study’s Steering Committee, we will develop a theory of change to model BTC program inputs, outputs, outcomes, and potential lifetime health and non-health impacts for children engaged in the intervention. Using Microsoft Excel, we will develop a decision analytic model (e.g., decision tree) to link the program theory of change to an economic framework for analysis. We will then undertake a scoping review of the published and grey literature to identify studies and potential data sources that can be used to estimate the monetary value associated with multi-sector program outcomes and impacts (e.g., reduced adverse health and mental health outcomes, improved academic outcomes, and improved economic productivity). Next, we will estimate the costs of implementation of the BTC program based upon the resources required for implementation, as outlined in our theory of change (inputs). Finally, we will combine the evidence on intervention program outcomes and impacts (benefits) and cost of implementation (costs) to yield a benefit-cost ratio, which will provide an estimate of the broader socio-economic returns to society for every dollar invested in BTC. Where there is uncertainty in estimates (e.g., the monetary value that can be attributed to a specific outcome), we will conduct sensitivity and threshold analyses to determine the impact of varying these estimates over a range of acceptable values on the overall SROI calculation. All costs and benefits that are incurred beyond one year will be discounted at an annual rate of 1.5%, in line with Canadian guidelines [21], to estimate the net present value of the SROI [54].

### Sample size and power calculations

The study is powered to detect a between-group effect size of Cohen’s d = 0.70 in the primary outcome (change in ASQ-3 between baseline to 24-month post-engagement) for research question 1a. This estimated effect size is conservative compared to the effect size reported in a similar study [55]. Using G*Power v3.1.9.7 [24], assuming a two-sided two-sample t-test with un-pooled variance, we estimate that *N* = 98 participants will be required (*n* = 49 per group). Assuming ~ 30% participant attrition [25], a sample size of *N* = 68 participants with complete data (*n* = 34 per group) achieves 81.1% power (a = 0.05) to detect between-group differences in the primary outcome. Thus, *n* = 50 per group will be sufficient.

### Data monitoring

There will be no data monitoring committee, interim analyses, or auditing for the study as it evaluates behavioural interventions that are currently being offered. Should a participant experience any adverse events or other unintended effects, these will be recorded by the clinicians and CBR and managed following regular procedures. Any scores that are considered critical or at-risk, particularly for the CES-D and BAI, will warrant a follow-up discussion and recommendation for the mother to seek further services. The CBRs may suggest that the participant contact their healthcare provider and provide a list of mental health resources if needed.

### Ethics and dissemination

Ethics approval has been obtained from the University of Ottawa Research Ethics Board (REB # H-02-23-8940) and all participants will provide written consent to participate in the research, to be contacted for follow-ups, and for researchers to access their clinical files. Participation in the research is voluntary and mothers will be able to receive services at BTC or MWCHC regardless of their desire to participate. Mothers at BTC and MWCHC will be made aware that they are under no obligation to participate and that if they choose to participate, they are able to withdraw from the study at any time and refuse to answer any questions without suffering any consequence. They will also have the option to take breaks while answering the questionnaires and will be provided with a list of current resources in their area should they experience any distress. BTC and MWCHC clinicians will be available to debrief with participants and support them as needed. In appreciation for their time and efforts, mothers will receive food vouchers for $50 at baseline, $75 at 12 months, and $100 at 24 months. The amounts increase to reflect the increased value of additional data points, to compensate for challenges that mothers may have in maintaining contact with the research group, and to reduce attrition. Mothers who are no longer engaged in services but participate in the research will also receive the compensation.

The results of the study will be communicated and disseminated within scientific and clinical services communities. Dissemination outputs will include fact sheets, research briefs, research conference poster and oral presentations, as well as scientific articles submitted to international peer-reviewed journals. The authors will consist of members of the research team who have made significant contributions to the study design, data collection and analysis, and manuscript writing. Participants’ identities will not be revealed in any publications resulting from this study.

## Discussion

This study is the first to examine the effectiveness of infant mental health components of a mother-child relationship-focused intervention program on infant mental health outcomes as compared to a mother-focused program. It aims to establish the comparative effectiveness and mechanisms of change of the infant mental health components of BTC, as well as estimate the long-term SROI of the integrated BTC approach. The involvement of MWCHC participants as a control group is a considerable strength of the study, thus making it possible to attribute changes in infant mental health outcomes to the enhanced BTC intervention. Although non-randomized studies suffer from an inherent selection bias, randomization is not possible due to feasibility and ethical reasons. Analytic approaches will be used to minimize any baseline differences between the BTC and MWCHC groups.

It is important to note that while the MWCHC program does not provide the same level of direct intervention with children as the BTC program, children do receive healthcare and well-baby visits. As well, MWCHC mothers receive basic parenting supports and mental health and addiction services that indirectly benefit their children. Their participation in this study will allow them to receive a summary report after every time point, containing an overview of their results as well as a developmental report for their child that they would not have otherwise received. Among other benefits for MWCHC participants, the implementation of the clinical measures will allow for better screening and referral and will increase evidence-based knowledge provided to clinicians. The introduction and embedment of a CBR will also enhance the capacity for research at each site, thus increasing the quality of regular clinical services being offered.

With over 25 years of developing infant mental health programming, BTC is in a unique position to study the effectiveness of an infant mental intervention for children of substance-involved mothers. Canada lags behind other wealthy nations with regards to investments, policies, and practices that support early child mental health [19]. ACEs in infancy and early childhood set the stage for lifelong mental health difficulties, with a failure to intervene resulting in significant health and economic consequences [18]. In the aftermath of the COVID-19 pandemic, there is an urgent need to prioritize the mental health of the next generation. Bringing together a nationally acclaimed team, this study aligns with national strategies to establish strong evidence for infant mental health interventions that reduce exposure to ACEs and support the mother-child relationship.

### Supplementary Information

**Additional file 1.** SPIRIT 2013 Checklist.

## Data Availability

Not applicable.

## Abbreviations

ACEs: Adverse Childhood Experiences
BTC: Breaking the Cycle
CBR: Community-based researcher
MWCHC: Maxxine Wright Community Health Centre
SROI: Social return on investment
